# Delivery of Cancer Survivorship Education to Community Healthcare Professionals

**DOI:** 10.1007/s13187-022-02164-w

**Published:** 2022-04-08

**Authors:** Ashley C. Pariser, Javin Brita, Maura Harrigan, Scott Capozza, Angela Khairallah, Tara B. Sanft

**Affiliations:** 1 Division of Medical Oncology, Wexner Medical Center, The Ohio State University, 1800 Cannon Drive, Lincoln Tower Suite 1300L, Columbus, OH 43210 USA; 2 Yale New Haven Hospital, PO Box 208028, New Haven, CT 06520-8028 USA; 3 Yale School of Public Health, 60 College St, New Haven, CT 06510 USA; 4 Yale New Haven Hospital, 20 York St, EP 10-635, New Haven, CT 06510 USA; 5 Section of Medical Oncology, Yale School of Medicine, PO Box 208028, New Haven, CT 06520-8028 USA

**Keywords:** Cancer survivorship, Continuing medical education, Supportive oncology, Project ECHO, Telehealth

## Abstract

Our pilot study aimed to evaluate the needs of community oncology providers with regard to cancer survivorship education, develop a survivorship curriculum based on the needs assessment, and evaluate the acceptability of the Project ECHO® (Extension for Community Healthcare Outcomes) model for delivery of the survivorship curriculum. A needs assessment was delivered to participants in suburban community cancer practices, and a curriculum was developed based on the results. Participants were enrolled in an ECHO curriculum consisting of 6 sessions from October to December 2019. Participants included registered nurses (RN), registered dietitians (RD), clinical social workers (LCSW), advanced practice providers (APP), radiation oncologists, and medical oncologists (MD). Participants were invited to participate in exit interviews designed to better evaluate the participant experience. Ninety percent of needs assessment participants (*n* = 37) expressed an interest in cancer survivorship education. Eight participants from 3 community practices in suburban Connecticut enrolled in the ECHO curriculum. Four participants (50%) agreed to participate in exit interviews. Five themes emerged from the exit interviews: interest in survivorship, time, positive experience, empowerment, and community. Our Survivorship ECHO pilot demonstrated the acceptability of the Project ECHO® model for delivering cancer survivorship education to oncology providers. Further research confirming the feasibility of this model in additional oncology provider settings is needed.

## Introduction

One in three individuals is projected to be affected by cancer in their lifetime. As cancer care and treatments improve, the number of cancer survivors is anticipated to grow from 15 to 19 million by 2024 [1]. Cancer survivors have distinct medical needs including ongoing primary and secondary cancer surveillance, management of treatment-related conditions, and psychosocial support with 30 to 50% of survivors indicating at least one unmet need [2]. There is an established correlation between survivors with unmet needs and decreased adherence to treatment and surveillance as well as worse perceived mental and physical health [3]. Disparities have been well documented with those from underserved and minority populations having inequitable access to quality survivorship care [4]. Access is further limited given the need for provider training and a dearth of available educational resources [5].

Cancer survivorship care is an integral part of oncology care. Oncologists overwhelmingly demonstrate a desire to be a part of their cancer survivors’ care, and 40% of oncologists do not feel comfortable providing general health maintenance, screening, and prevention care [6]. To optimize care for their survivors, these providers both desire and need high-quality continuing education focused on survivorship care.

In 2006, the Institute of Medicine’s (IOM) sentinel publication “From Cancer Patient to Cancer Survivor: Lost in Transition” identified the need for educational opportunities for health providers as one of its ten recommendations [7]. Progress has been made with the development of survivorship guidelines by the American Society of Clinical Oncology (ASCO), National Comprehensive Cancer Network (NCCN), and American Cancer Society (ACS); however, there is currently no standard of care for survivorship care education.

Project ECHO® utilizes telehealth to promote long-distance learning and sharing of best practices. Learning loops are established utilizing short didactics, case-based discussions, and formative feedback [8]. The feasibility of the Project ECHO® model to train community providers to provide specialized care has been demonstrated in numerous studies [9]. Multiple studies evaluating individual ECHOs have demonstrated changes in the provider’s knowledge, improvements in provider competence, and the potential to improve patient outcomes. Currently, there are 35 active Project ECHO® sites in the USA focusing on cancer prevention, surveillance, or treatment including 13 focusing on survivorship care [2]. We are the first pilot study to evaluate the acceptability of utilizing the Project ECHO® platform to educate community oncology providers on survivorship best practices.

## Methods

Our pilot study set out to accomplish three aims: (1) perform a community-based needs assessment; (2) develop a survivorship curriculum based on the results of the needs assessment; (3) evaluate the acceptability of the Project ECHO® model for delivery of the survivorship curriculum.

Our urban academic cancer center is affiliated with fourteen community-based oncology practices. We recruited participants from four of these practices through email and onsite presentations. Eligible participants included licensed and actively practicing MD, APP, RN, LCSW, and RD. Trainees who did not exclusively practice at a community oncology practice within the Yale Cancer Network were excluded.

We developed our needs assessment based on ASCO guidelines, NCCN guidelines, and our team’s over 14 years of experience and aligned the assessment with the four tenets of survivorship care: surveillance, managing late and long-term effects of treatment, encouraging healthy lifestyle behaviors, and coordination of care [6, 10]. The needs assessment was administered during on-site visits to four local community-based oncology practices in Connecticut.

The second aim was to develop a cancer survivorship curriculum based upon the results of the needs assessment. The curriculum was developed based on the results of the needs assessment and included six lectures with case-based discussion, a brief didactic, and a focused, facilitated discussion. The curriculum was developed and delivered by our multidisciplinary Survivorship ECHO team consisting of an oncologist (MD), physician assistant (PA-C), RD, physical therapist (PT), and LCSW. Each session was planned to last 1 h and be delivered over Zoom technology. The cancer survivorship curriculum was delivered over a 3-month time frame.

The third aim was to evaluate the acceptability, appropriateness, and feasibility of the Project ECHO model for survivorship educational delivery to community oncology providers [11, 12]. All participants were invited to complete post-session surveys, summative end-of-course surveys, and to email with questions and feedback. Additionally, all ECHO participants were sent email invitations to participate in a one-on-one exit interview upon completion of the survivorship ECHO.

Exit interviews were designed to better evaluate the participant experience as well as the acceptability of the ECHO platform for the delivery of cancer survivorship education. Questions included in the semi-structured exit interviews are shown in Table 1. In-person and telephone exit interviews were completed and transcribed by the author (AP). All interviews were conducted between interviewer and participant in a setting of the participant’s choosing. Field notes were taken at the conclusion of each interview and utilized for data processing. Inductive data analysis was conducted by the first author. Field notes were shared with all authors who confirmed identified themes. Themes were selected due to the commonality across interviews and field notes. All selected themes were present in at least 75% of the interviews with 4 of the 5 themes present in 100% of the interviews.

**Table 1:** Semi-structured exit interview questions

1. What were your barriers to joining ECHO?	7. What do you recommend for improving future sessions?
2. How would you describe your experience?	8. Would you recommend the ECHO platform to your colleagues?
3. What were your barriers to joining individual sessions?	9. Would you recommend our Survivorship ECHO to your colleagues?
4. What did you gain from the experience?	10. Would you be interested in future sessions?
5. Did the sessions influence your practice (facility)?	11. What additional topics would you be interested in?
6. Did the sessions influence your clinical care?	12. Anything else you would like to discuss?

Our pilot study focused on stage I of the NIH Stage Model for Behavioral Intervention Development, which calls for refinement, adaptation, and pilot testing of an intervention with the ultimate goal of highly implementable and potent interventions that can be applied and studied in diverse real-world settings. This is the first pilot to evaluate the acceptability of utilizing the Project ECHO® model to educate community-based oncology providers [13].

## Results

### Needs Assessment

The needs assessment for our pilot study was conducted at 4 community practice sites in our cancer network during regularly scheduled monthly staff meetings that included providers from multiple disciplines (*n* = 42). Ninety percent of participants (*n* = 37) expressed an interest in cancer survivorship education. A high level of interest was expressed in the following topics: transitioning patients from active treatment to survivorship, fear of recurrence, weight management, anxiety and depression counseling, management of fatigue, management of peripheral neuropathy, and management of cognitive impairment (Table 2). Participants expressed the least amount of interest in managing survivorship guilt, management of cardiotoxicity, and complementary medicine.

**Table 2:** Needs assessment of potential survivorship education topics

Survivorship topic	Interested	Not interested	Percent interested
Adjusting to survivorship	22	6	79%
Anxiety and depression	14	4	78%
Cancer genetics	16	13	55%
Cardiotoxicity	14	16	47%
Cognitive impairment	25	5	83%
Complementary medicine	10	7	41%
Fatigue	26	4	87%
Infertility and pregnancy	27	10	73%
Myths about nutrition	20	10	67%
Neuropathy	27	3	90%
Nutritional supplements	19	10	65%
Osteoporosis	11	8	58%
Survivor guilt	9	21	30%
Risk of recurrence	20	7	74%
Weight management	20	10	67%

While conducting the needs assessment, not all participants answered all questions. Participants who responded yes or no for each question have been included in the table above

### Curriculum Development and Delivery

Based on the results of the needs assessment, the curriculum was formulated around sessions with the following themes: survivorship 101, fear of recurrence, energy balance, fatigue, and late effects of treatment. The final session was reserved for the topic of the highest interest of confirmed participants to account for potential differences in the educational needs of Survivorship ECHO participants compared to needs assessment participants. Based on the preferences of the participants, an additional session on sexual dysfunction was developed. Session titles and learning objectives can be seen in Table 3.

**Table 3:** Curriculum learning objectives

Lecture	Learning objectives
**Survivorship 101**	Define survivorship and survivorship phases
	Review survivorship epidemiology
	Describe common concerns of survivors
	Summarize the role of the multidisciplinary team
**Fear of recurrence**	Define fear of cancer recurrence
	Identify levels of fear of recurrence and patients most vulnerable to developing a fear of recurrence
	Summarize strategies for managing fear of recurrence
**Energy balance**	Identify the role of nutrition, physical activity, and psychosocial perspective on energy balance
	Summarize strategies for optimizing survivors’ energy balance
**Fatigue**	Summarize screening and assessment strategies for cancer-related fatigue
	Summarize management strategies for cancer-related fatigue
**Late effects of treatment**	Identify causes of peripheral neuropathy
	Identify causes of lymphedema
	Describe assessment strategies for peripheral neuropathy
	Describe assessment strategies for lymphedema
	Summarize management strategies for peripheral neuropathy
	Summarize management strategies for lymphedema
**Sexual dysfunction**	Summarize sexual concerns of survivors
	Identify causes of sexual dysfunction
	Describe available treatments for sexual dysfunction

Of the four community oncology practices where the needs assessment was performed, three had representatives who participated in the ECHO. Of the 37 who indicated an interest in a Survivorship ECHO, 8 (22%) enrolled to participate. Disciplines represented in the enrollees included MD, APP, LCSW, and RNs. There were no trainee participants. Reasons for participants who had an initial interest during the needs assessment but declined enrollment included clinical responsibilities, lack of protected time, the timing of sessions, and inadequate conference space and technology. All participants who expressed interest in participating in our Survivorship ECHO were emailed invitations to participate. Barriers described above were described in email responses from participants. Unfortunately, due to the COVID-19 pandemic, a formal assessment of barriers to enrollment for needs assessment participants who described interest in enrollment but ultimately did not enroll was unable to be performed. Over the course of 6 sessions, participation averaged 6 participants per session (75%).

Content and structure were modified after the initial post-session feedback identified difficulty accessing the video conferencing capabilities of the Zoom platform. This led to the distribution of slides prior to each session to enhance accessibility.

### Acceptability Analysis

Post-session survey completion was 15% (*n* = 7/48), and post-ECHO summative survey completion was 25% (*n* = 2/8). Several positive themes arose from these surveys, including a collegial and welcoming learning environment and a desire for summarized best practices. Constructive feedback included suggestions of spending less time on didactics and more time on case-based discussions. Given low rates of survey completion, a semi-structured interview was developed to explore the acceptability of the Project ECHO model for the delivery of survivorship education.

Of the eight ECHO participants, four (50%) agreed to participate in semi-structured exit interviews: 2 MDs, 1 LCSW, and 1 APP. From these interviews, five central themes emerged (Table 4).

**Table 4:** Exit interview themes

Interest in survivorship	Time demands	Comprehensiveness	Empowerment	Community
Main motivator to participate	The biggest barrier to participation	Well-structured and organized curriculum	Increased awareness of cancer survivors’ needs	Opportunity to network
Interested in best practices	Time required and investment was doable	Comprehensive review	Increased comfort addressing survivorship topics	Decreased sense of isolation

The first theme expressed by all four participants was an interest in survivorship. This served as the primary motivator to participate in the Survivorship ECHO. The ECHO curriculum served as an opportunity for a refresher on survivorship best practices. Survivorship was described by all four participants as applicable to their clinical practice; however, self-instruction and review were limited given competing clinical and educational demands.

Representative time demands quote:

*“I don’t have time to read up on the latest literature.* [Survivorship] *gets pushed to the bottom of the list particularly when more urgent concerns like ‘my patient is progressing’ comes up”*

The second theme that emerged was time demands. Two participants described difficulties staying connected to colleagues and accessing other continuing education opportunities from their community practice locations. Time away from clinical responsibilities as well as driving to remote CME activities were noted as barriers. ECHO helped address those concerns, as two participants identified the remote learning opportunity as “doable” with minimal time commitment and disruption to patient care responsibilities.

The third theme was the comprehensiveness of the curriculum. The curriculum was described as a “good review” with a “nice progression,” “very organized,” and “plenty of opportunities for questions.” Augmentation of their provider toolkit and improved knowledge of survivorship resources was noted by all respondents. Additionally, all respondents would recommend both the ECHO format as well as our Survivorship ECHO to their colleagues.

The fourth theme identified was empowerment. Two providers described significant changes in their clinical practice associated with both a sense of empowerment and confidence to address the concerns of survivors. All providers described improved access to resources for their survivors and increased comfort in referring patients to survivorship clinics.

Representative empowerment quote:

*“I’m doing it. I never would have asked before. For example, I saw a newly diagnosed breast cancer patient and found out that she had a fear of recurrence. I wouldn’t have even asked before, but now I ask clarifying questions and was able to provide counseling, reassurance and offer survivorship clinic as a resource.”*

The fifth and last theme to emerge was an enhanced sense of community. All four participants appreciated the opportunity to engage with colleagues from different specialties and practice locations.

Representative engagement quote:

*“I felt a part of the [Network] Cancer Care Team. I work in a standalone center without radiation, labs, medical oncology, anyone else. No cafeteria even. It was great even on the phone to connect with colleagues. Loved being able to learn from other APPs, MDs”.*

The identified barriers to participation in our pilot study were time and technology. One participant had never utilized Zoom prior to participation in the Survivorship ECHO. Additionally, most participants did not have access to a webcam or conference room with webcam capabilities. As a result, most participants called into sessions, minimizing face-to-face interactions between participants. Both MDs described difficulties rescheduling patients and clinical responsibilities as barriers to joining individual sessions. All participants recommended greater notice prior to implementation of ECHO sessions so that schedules could be modified to allow for enhanced participation.

## Discussion

There are currently millions of cancer survivors in the USA, and the number is expected to continue to increase substantially in the coming decades. Cancer survivors are a distinct population with multifaceted needs [1]. These needs, however, have historically been unmet and physicians continue to express barriers to addressing the concerns of cancer survivors including time constraints as well as a lack of both expertise and resources [14–16].

Currently, survivorship education is being delivered in a myriad of formats with the vast majority prior to the COVID-19 pandemic being delivered as in-person conferences or symposiums [7]. Limited data exists evaluating the needs of community oncology providers. Schwartz et al. demonstrated interest among pediatric residents for cancer survivorship care, and several publications have demonstrated both a need and desire among nurses for formalized cancer survivorship education [17–19]. Although needs assessments targeting medical oncologists are lacking in the literature, medical oncologists and primary care physicians self-reported a lack of formalized training as a barrier to the delivery of survivorship care [20].

Our findings add to a limited body of literature describing the needs of oncology providers for survivorship education and propose an acceptable format with the potential to deliver curriculum to a multidisciplinary audience. Overall, data available demonstrates a desire amongst providers for greater access to cancer survivorship educational opportunities [17–19, 21]. This was validated by our needs assessment with 90% of our participants expressing interest in cancer survivorship education.

Currently, there is no standard of care with regard to survivorship training and education. Prior exposure to formal training varies significantly depending upon specialty as well as when providers received their formal training. The field of survivorship remains a burgeoning field with numerous advances and changes over the last 2 decades. Furthermore, survivors engage with multiple providers within the field of oncology as part of their treatment trajectory. At our institution, any provider can refer a patient to our specialty cancer survivorship clinic. Thus, embedded in our second aim was a goal of enhancing consistent, guideline-based communication between all healthcare personnel and survivors. This involved a three-prong approach:

1. Review and reinforce common and guideline-based survivorship care principles.
2. Promote specialized knowledge in core areas of survivorship.
3. Identify concerns that would benefit from referral to a specialized survivorship clinic.

Thus, we recruited providers of all clinical backgrounds as participants in our Survivorship ECHO.

We were able to enroll providers from multiple disciplines including physicians, advanced practice providers, registered nurses, and clinical social workers in three community practices in suburban Connecticut. The strengths of our Survivorship ECHO included a comprehensive, multidisciplinary curriculum based on both a needs assessment and national guidelines. Additionally, the semi-structured exit interviews demonstrated the opportunity of the ECHO platform to enhance individual provider competency and confidence. Although our sample number is small, two of our providers (50%) acknowledged a meaningful change in their practice, and all four providers described an increased toolkit and awareness of resources available to their cancer survivors. Additionally, providers who participated in our semi-structured exit interviews described an enhanced sense of community and decreased sense of isolation that has been demonstrated in other studies evaluating the Project ECHO® model [22]. This unintended consequence may prove to be even more desirable as we continue to practice in the context of a global pandemic.

There are several limitations of our study. First, participants were intrinsically interested in survivorship care and likely reflect a highly motivated, self-selected population. Additionally, most participants were not provided with protected time to participate. This likely contributed to a significantly smaller enrollment when compared to interest in participation demonstrated in our needs assessment and may have contributed to low post-session survey completion. Furthermore, we recruited a diverse population of participants from multiple specialties with differing time commitments and availability. For instance, although most needs assessment participants were nurses, most pilot participants were not nurses. Thus, the availability of clinical nurses likely differs from the availability of physicians. Diverse recruitment may have had the unintended consequence of deterring participants who feel more comfortable learning in settings dedicated to their specific specialty. Thus, implementing a structured assessment of barriers for participants and nonparticipants of future ECHO offerings will be important for continued program development. Conversely, enrolled participants found the multidisciplinary aspect of the sessions including learning from both a multidisciplinary panel of experts and participants to be one of the strengths of the program. The difference between rates of interest and rates of participation highlights the real-world challenges of establishing new educational programs in the community setting. Importantly, it also reflects a need to engage important clinic and organizational stakeholders in the design, implementation, and assessment of program development.

The biggest limitation of our pilot study is our small sample size. Although we identified more than 30 interested participants, we had 8 participants enroll in the pilot ECHO sessions and 4 participate in exit interviews. Our sample size limits our ability to confirm the feasibility of the Project ECHO model for the delivery of cancer survivorship education; however, the model was well received and acceptable to all enrolled participants. We utilized the Project ECHO® model to deliver a Cancer Survivorship curriculum providing our participants with an educational program that was inclusive of the 6 core competencies developed by the American Board of Medical Specialties (ABMS)—particularly interpersonal and communication skills, systems-based practice, patient care, and medical knowledge [8]. Feedback during sessions as well as via post-session surveys and exit interviews highlighted that the Project ECHO model was an acceptable platform for delivery of the curriculum. Our experience highlights the opportunity of telehealth and teleconferencing to meet the educational needs of providers irrespective of the providers’ practice location. Future directions include broadening the audience to enrollees providing survivorship care in other areas across the country and internationally.

Our pilot study utilized a low-cost, multidisciplinary platform to educate community oncology providers in a needs-based cancer survivorship educational curriculum. Given disparities in survivorship care, there is a need for alternative delivery of continuing education and training of providers to expand access to this care. Further research evaluating the acceptability of the Project ECHO model for delivering survivorship education in additional oncology practice settings is needed.
